# Galectin-3 critically mediates the hepatoprotection conferred by M2-like macrophages in ACLF by inhibiting pyroptosis but not necroptosis signalling

**DOI:** 10.1038/s41419-022-05181-1

**Published:** 2022-09-08

**Authors:** Li Bai, Wang Lu, Shan Tang, Huixin Tang, Manman Xu, Chen Liang, Sujun Zheng, Shuang Liu, Ming Kong, Zhongping Duan, Yu Chen

**Affiliations:** 1 The Fourth Department of Hepatology, Beijing YouAn Hospital, Capital Medical University, 100069 Beijing, China; 2 Beijing Municipal Key Laboratory of Liver Failure and Artificial Liver Treatment Research, 100069 Beijing, China; 3 The First Department of Hepatology, Beijing YouAn Hospital, Capital Medical University, 100069 Beijing, China

**Keywords:** Inflammasome, Necroptosis

## Abstract

We previously documented that M2-like macrophages exert a hepatoprotective effect in acute-on-chronic liver failure (ACLF) by inhibiting necroptosis signalling. Nevertheless, the molecular mechanism behind this hepatoprotection still needs to be further dissected. Galectin-3 (GAL3) has been reported to be critically involved in the pathogenesis of multiple liver diseases, whereas the potential role of GAL3 in ACLF remains to be explored. Herein, we hypothesised that GAL3 plays a pivotal role in the hepatoprotection conferred by M2-like macrophages in ACLF by inhibiting necroptosis. To test this hypothesis, we first assessed the expression of GAL3 in control and fibrotic mice with or without acute insult. Second, loss- and gain-of-function experiments of GAL3 were performed. Third, the correlation between GAL3 and M2-like macrophage activation was analysed, and the potential role of GAL3 in M2-like macrophage-conferred hepatoprotection was confirmed. Finally, the molecular mechanism underlying GAL3-mediated hepatoprotection was dissected. GAL3 was found to be obviously upregulated in fibrotic mice with or without acute insult but not in acutely injured mice. Depletion of GAL3 aggravated hepatic damage in fibrotic mice upon insult. Conversely, adoptive transfer of GAL3 provided normal mice enhanced resistance against acute insult. The expression of GAL3 is closely correlated with M2-like macrophage activation. Through adoptive transfer and depletion experiments, M2-like macrophages were verified to act as a major source of GAL3. Importantly, GAL3 was confirmed to hold a pivotal place in the hepatoprotection conferred by M2-like macrophages through loss- and gain-of-function experiments. Unexpectedly, the depletion and adoptive transfer of GAL3 resulted in significant differences in the expression levels of pyroptosis but not necroptosis signalling molecules. Taken together, GAL3 plays a pivotal role in the hepatoprotection conferred by M2-like macrophages in ACLF by inhibiting pyroptosis but not necroptosis signalling. Our findings provide novel insights into the pathogenesis and therapy of ACLF.

## Introduction

Acute-on-chronic liver failure (ACLF) occurs in patients with preexisting chronic liver diseases and is usually associated with a precipitating event [1–3]. Although decades of efforts made by clinicians and researchers promote our understanding of the pathogenesis and therapy of ACLF, there is still an urgent need to dissect the cellular and molecular mechanisms of ACLF and to seek new targets for ACLF therapy.

In recent years, we have endeavoured to probe the pathogenesis of ACLF. Through in vivo and in vitro experiments, we have demonstrated the following: (1) hepatic fibrosis protects mice from diverse lethal insults; (2) the hepatoprotection conferred by hepatic fibrosis can be attributed to M2-like macrophages; (3) in terms of cellular mechanism, M2-like macrophages protect hepatocytes against apoptosis and (4) the phenotypic switch of human and mouse macrophages (towards the M2-like phenotype) confers enhanced resistance to apoptosis to hepatocytes [4–6].

Recent advances in the field of cell death have placed a new emphasis on regulated necrosis [7, 8]. As an important component of regulated necrosis, necroptosis has aroused wide concern from clinical physicians and researchers. However, the precise role of necroptosis in liver injuries remains controversial [9, 10]. In the case of acetaminophen (APAP)-induced acute liver injury, Dara et al. [11] believed that receptor-interacting serine/threonine-protein kinase (RIPK) 1 mediates murine APAP toxicity independent of the necrosome and not through necroptosis; however, several investigators have reported that RIPK3 KO protects mice against APAP toxicity [12, 13]. Very recently, we integrated necroptosis signalling into the hepatoprotection mediated by M2-like macrophages occurring in ACLF and documented that M2-like macrophages confer beneficial protection against acute insult in mice by inhibiting the necroptosis-S100A9–necroinflammation axis [14]. Nevertheless, the specific molecular mechanism behind this finding still needs to be dissected.

Galectin-3 (GAL3), a β-galactoside-binding lectin, has been demonstrated to be involved in a wide range of biological processes, such as regeneration, cell migration, inflammatory and immune responses [15, 16]. To date, the role of GAL3 in multiple human diseases is still a contentious issue. Recently, AI-Salam et al. [17] have identified GAL3 as an anti-apoptotic mediator in cardiomyocytes at 24 h post-myocardial infarction, and they further demonstrated that high GAL3 after myocardial infarction has a protective role on the heart through modulating lysosomal Cathepsins in ischaemic myocardium [18]. However, other studies have shown that GAL3 interferes with tissue repair and promotes cardiac dysfunction and comorbidities [19], and elevated GAL3 is associated with an increased risk of death and heart failure (HF) [20]. Regarding hepatic injury, GAL3 has been documented to suppress TNF-α-dependent hepatocyte death and liver inflammation in MCMV infection [21]. In contrast, Volarevic et al. confirmed that GAL3 plays an important proinflammatory role in ConA-induced hepatitis [22]. To date, only two reports (one Chinese work and one Abstract) by the same team have referred to the serum expression of GAL3 in ACLF. In view of this, it is imperative to investigate the possible role of GAL3 in our ACLF model and to further clarify the contribution of GAL3 to the hepatoprotection conferred by M2-like macrophages.

In this work, we investigated the interplay among M2-like macrophages, GAL3 and necroptosis signalling and hypothesised that GAL3 acts as a critical player in the hepatoprotection conferred by M2-like macrophages in ACLF by inhibiting necroptosis-S100A9-necroinflammation. For this aim, first, we determined the expression levels of GAL3 in control and fibrotic mice with or without acute challenge; second, we assessed the impact of GAL3 on hepatic damage in control or fibrotic mice upon insult through gain- and loss-of-function experiments; then, we analysed the correlation between GAL3 expression and M2-like macrophage activation; next, we attempted to verify the pivotal role of GAL3 in the hepatoprotection mediated by M2-like macrophages; and finally, we wanted to confirm the inhibitory effects of GAL3 on necroptosis-S100A9-necroinflammation through the depletion and adoptive transfer of GAL3.

## Results

### GAL3 is upregulated in fibrotic mice with or without acute insult but not in acutely injured mice

**Fig. 1:**
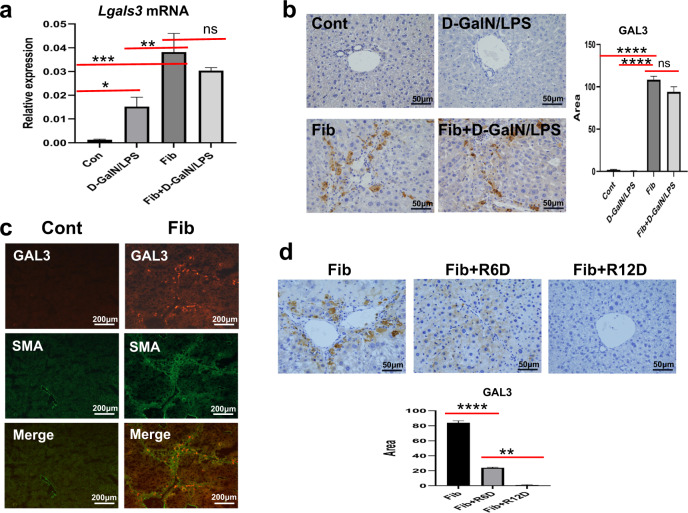
GAL3 is upregulated in the fibrotic liver, even under acute insult. **a** The mRNA levels of *Lgals3* in control and fibrotic mice with or without acute insult. **b** The protein expression of GAL3 in control and fibrotic mice with or without acute insult. **c** The colocalisation of GAL3 and SMA in the fibrotic liver. **d** The protein expression of GAL3 in the liver of regressive mice (*n* = 4–6). Group comparisons were performed using one-way ANOVA followed by Tukey’s multiple comparison test, *P < 0.05, **P < 0.01, ***P < 0.001, ****P < 0.0001.

First, we compared the expression levels of GAL3 in control and fibrotic mice with or without acute insult. As shown in Fig. 1a, the transcriptional levels of *Lgals3* assessed by quantitative PCR detection were much higher in fibrotic livers with or without D-GalN/LPS insult than in acutely injured livers (*P* < 0.01). Accordingly, the protein expression of GAL3 detected by IHC analysis was markedly enhanced in the livers of fibrotic mice, even under insult. Nevertheless, this was not the case in the livers of acutely injured mice (Fig. 1b). Moreover, GAL3 was found to colocalise with SMA, a representative marker of hepatic fibrosis (Fig. 1c). Furthermore, the expression of GAL3 was decreased in mice during the regression phase (Fig. 1d). Thus, GAL3 is significantly upregulated in the fibrotic setting, even under insult.

### GAL3 exerts favourable hepatoprotection against acute insult in control and fibrotic mice

**Fig. 2:**
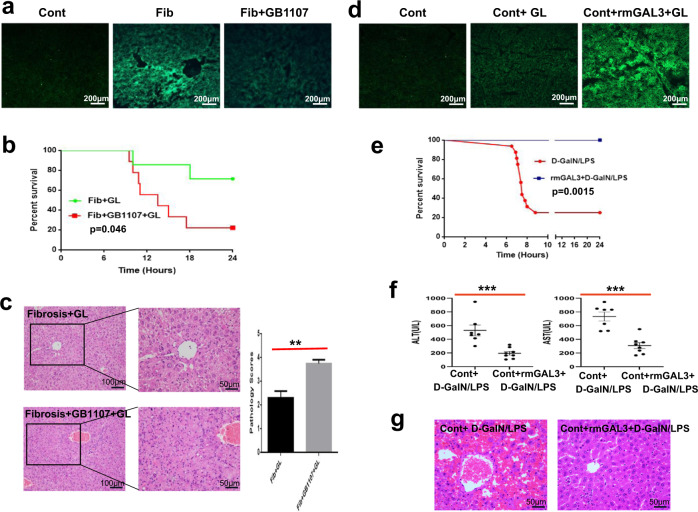
GAL3 exerts beneficial hepatoprotection against acute insult in control and fibrotic mice. **a** The expression of GAL3 detected by IF staining in fibrotic livers with or without GB1107 treatment. **b** Comparison of the survival rate in fibrotic mice upon insult with or without GB1107 treatment (*n* = 7–9). Survival analysis was conducted with the Kaplan–Meier method, and the log-rank test was used to compare the differences in survival between treatment groups. **c** Hepatic histology and pathology scores in fibrotic mice upon insult with or without GB1107 treatment (*n* = 8–13). Group comparisons were performed using the Mann–Whitney *U* test, **P < 0.01. **d** GAL3 expression in the livers of acutely injured mice with or without recombinant GAL3 pretreatment. **e** Comparison of the survival rate in acutely injured mice with or without recombinant GAL3 pretreatment (*n* = 8–16). The log-rank test was used to compare the differences in survival between treatment groups. **f** Serum ALT and AST levels in acutely injured mice with or without recombinant GAL3 pretreatment. Group comparisons were performed using the Mann–Whitney *U* test and Student’s *t* test, respectively, ***P < 0.001, ****P < 0.0001. **g** Hepatic histology in acutely injured mice with or without recombinant GAL3 pretreatment.

We previously documented that hepatic fibrosis confers enhanced resistance against diverse lethal insults, including D-GalN/LPS, in mice. In view of the obviously upregulated expression of GAL3 in the fibrotic liver and the colocalisation of GAL3 with SMA, we postulated that GAL3 may be responsible, at least in part, for the injury resistance occurring in fibrotic mice. To test this hypothesis, we depleted GAL3 in fibrotic mice with the specific inhibitor GB1107, followed by acute insult, and the hepatic damage was compared in fibrotic mice with or without GB1107 treatment. As expected, GB1107 treatment resulted in a marked downregulation of GAL3 in the livers of fibrotic mice (Fig. 2a). Regarding the survival rate, 7 of 9 (77.8%) fibrotic mice died in response to GB1107 treatment plus D-GalN/LPS challenge, whereas 2 of 7 (28.6%) fibrotic mice died upon challenge (*P* = 0.046) (Fig. 2b). Moreover, GB1107 treatment significantly aggravated hepatic histology in fibrotic mice in response to acute insult, which was assessed by H&E staining and pathology scores (Fig. 2c). The above-mentioned findings support the pivotal role of GAL3 in injury resistance in the setting of hepatic fibrosis.

To further confirm the beneficial hepatoprotection derived from GAL3, recombinant mouse GAL3 was adoptively transferred into normal mice via intraperitoneal injection, followed by acute insult. The survival rate and the extent of hepatic damage were compared in acutely injured mice with or without recombinant GAL3 pretreatment. As a result, adoptive transfer of recombinant GAL3 led to obvious upregulation of GAL3 in the livers of acutely injured mice (Fig. 2d). In terms of survival rate, 12 of 16 (75%) mice died in response to D-GalN/LPS challenge, whereas no deaths (0%) occurred in mice receiving recombinant GAL3 pretreatment plus acute insult (*P* = 0.0015) (Fig. 2e). Moreover, acute insult triggered sharp increases in serum ALT and AST levels in control mice; however, GAL3 pretreatment brought about obvious reductions in these two indices in acutely injured mice (both *P* < 0.001) (Fig. 2f). In line with this, the hepatic architecture was significantly improved in those mice receiving recombinant GAL3 (Fig. 2g). Therefore, GAL3 exerts beneficial hepatoprotection against acute insult.

### The expression of GAL3 is closely correlated with M2-like activation of macrophages

**Fig. 3:**
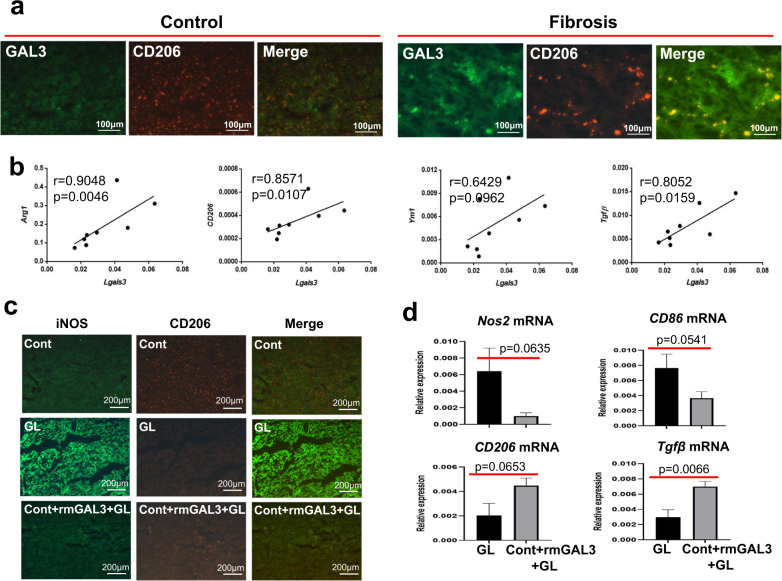
GAL3 expression is closely correlated with M2-like macrophage activation. **a** The colocalisation of GAL3 and CD206 in the fibrotic liver. **b** The correlation between *Lgals3* and M2-like macrophage activation markers, including *Arg1*, *CD206*, *Ym1* and *Tgfβ*. **c** The expression of the M1 marker iNOS and the M2 marker CD206 in the livers of acutely injured mice with or without recombinant GAL3 pretreatment. **d** The expression of the M1 markers (*Nos2* and *CD86*) and the M2 markers (*CD206 and Tgfβ*) in the livers of fibrotic mice upon insult with or without GAL3 depletion (*n* = 4–7). Group comparisons were performed using the Mann–Whitney *U* test or Student’s *t* test.

We previously demonstrated that injury resistance occurring in the fibrotic setting can be ascribed to M2-like macrophages. Herein, we wanted to explore whether the hepatoprotective effect exerted by GAL3 is also attributed to M2-like activation of macrophages. For this purpose, the correlation between GAL3 expression and M2-like macrophage activation was analysed. Immunostaining revealed that GAL3 was colocalised with CD206, the canonical marker of M2 macrophage activation, supporting the close association between GAL3 and M2-like macrophages (Fig. 3a). In particular, the gene expression of *Lgals3* exhibited positive relationships with those of *Arg1*, *CD206*, *Ym1* and *Tgfβ* (*P* = 0.0046, *P* = 0.0107, *P* = 0.0962 and *P* = 0.0159, respectively) in the livers of fibrotic mice (Fig. 3b). Moreover, markedly reduced iNOS but enhanced CD206 expression was noted in the livers of mice receiving recombinant GAL3 pretreatment (Fig. 3c). Meanwhile, upregulated M2 markers, including *CD206* and *Tgfβ*, but downregulated M1 markers, such as *Nos2* and *CD86*, were observed in GAL3-treated mice upon insult (Fig. 3d). Collectively, GAL3 is closely associated with M2-like activation of macrophages.

### M2-like macrophages act as a major cell source of GAL3

**Fig. 4:**
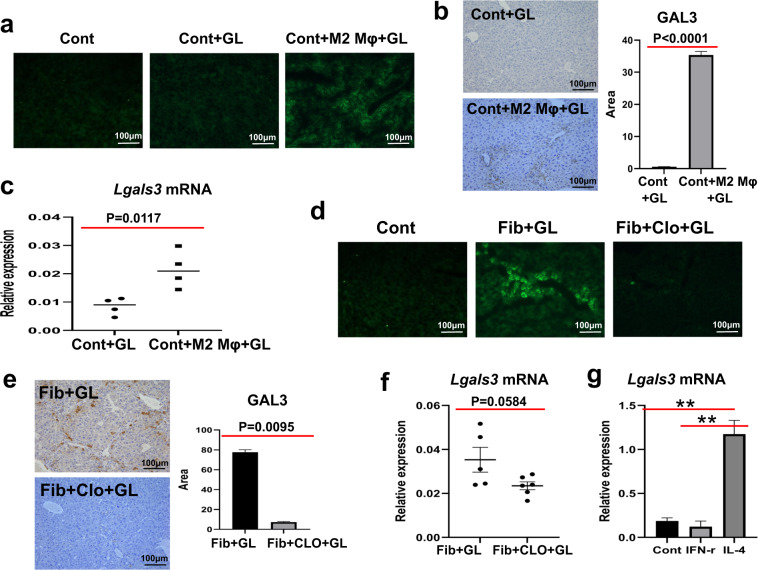
M2-like macrophages act as a key regulator of GAL3 expression. **a**, **b** GAL3 expression in acutely injured mice with or without M2-like macrophage transfer. **c** The mRNA levels of *Lgals3* in the livers of acutely injured mice with or without M2-like macrophage transfer. **d**, **e** GAL3 expression in fibrotic mice upon insult with or without M2-like macrophage depletion. **f** The mRNA levels of *Lgals3* in the livers of fibrotic mice upon insult with or without M2-like macrophage depletion. **g** The mRNA levels of *Lgals3* in M0, M1 or M2 macrophages (**a**–**c**: *n* = 4; **d**–**f**: *n* = 4–6; **g**: *n* = 3). Group comparisons were performed using Student’s *t* test, the Mann–Whitney *U* test, or one-way ANOVA followed by Tukey’s multiple comparison test, **P < 0.01.

To provide further evidence for the probable relationship between M2-like macrophages and GAL3, we adoptively transferred M2-like macrophages into normal mice by tail vein injection, followed by D-GalN/LPS challenge. Immunostaining analysis revealed that the hepatic expression of GAL3 was boosted in normal mice subjected to M2-like macrophage transfer and acute insult compared to that in acutely injured mice (Fig. 4a, b). Consistent with this finding, the mRNA levels of *Lgals3* were also significantly elevated in the livers of M2-like macrophage-transferred mice (Fig. 4c). Conversely, when we depleted M2-like macrophages in the fibrotic liver followed by acute insult, the gene and protein levels of GAL3 were sharply decreased (Fig. 4d–f). These results support that GAL3 may be derived from M2-like macrophages. We also performed an in vitro experiment to verify this finding. As a result, the expression of *Lgals3* was markedly enhanced in M2-like macrophages stimulated by IL-4 compared to that in M0 or M1 macrophages (Fig. 4g). Hence, M2-like macrophages seem to be a major cell source of GAL3.

### GAL3 holds a pivotal place in the hepatoprotection conferred by M2-like macrophages

**Fig. 5:**
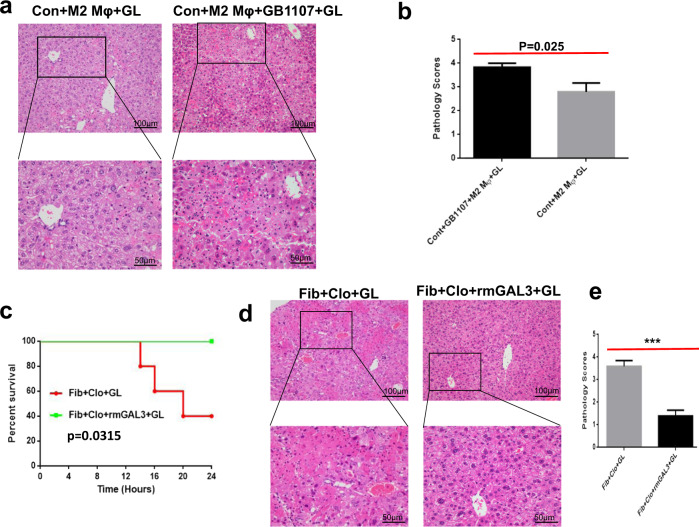
GAL3 holds a pivotal place in the hepatoprotection conferred by M2-like macrophages. **a**, **b** Hepatic histology and pathology scores in acutely injured mice subjected to M2-like macrophage transfer with or without GB1107 treatment (*n* = 5–6). Group comparisons were performed using Student’s *t* test. **c** Comparison of the survival rate in fibrotic mice upon macrophage depletion and acute insult with or without recombinant GAL3 treatment (*n* = 5–6). The log-rank test was used to compare the differences in survival between treatment groups. **d**, **e** Hepatic histology and pathology scores in fibrotic mice upon macrophage depletion and acute insult with or without recombinant GAL3 treatment (*n* = 5). Group comparisons were performed using Student’s *t* test, ***P < 0.001.

On the basis of the aforementioned findings, we speculated that GAL3 may hold a pivotal place in the hepatoprotection conferred by M2-like macrophages. To provide powerful support for this speculation, we treated normal mice with M2-like macrophages and GB1107 simultaneously, followed by acute insult. Interestingly, the histopathology was conspicuously deteriorated in mice upon M2-like macrophage and GB1107 treatment compared to that in mice receiving M2-like macrophage treatment only (Fig. 5a). The pathology scores assessed by experienced pathologists were higher in mice upon treatment with M2-like macrophages plus GB1107 (Fig. 5b). Hence, the administration of GB1107 destroyed the beneficial hepatoprotection conferred by M2-like macrophages. In contrast, when we adoptively transferred recombinant mouse GAL3 into fibrotic mice subjected to depletion of M2-like macrophages, followed by acute insult, hepatic injury was markedly mitigated, as evidenced by enhanced survival (100% vs. 40%, *P* = 0.0315) (Fig. 5c) and improved liver architecture (Fig. 5d, e). Accordingly, GAL3 holds a pivotal place in the hepatoprotection conferred by M2-like macrophages.

### Recombinant GAL3 confers hepatoprotection to acutely injured mice by suppressing pyroptosis but not necroptosis signalling

**Fig. 6:**
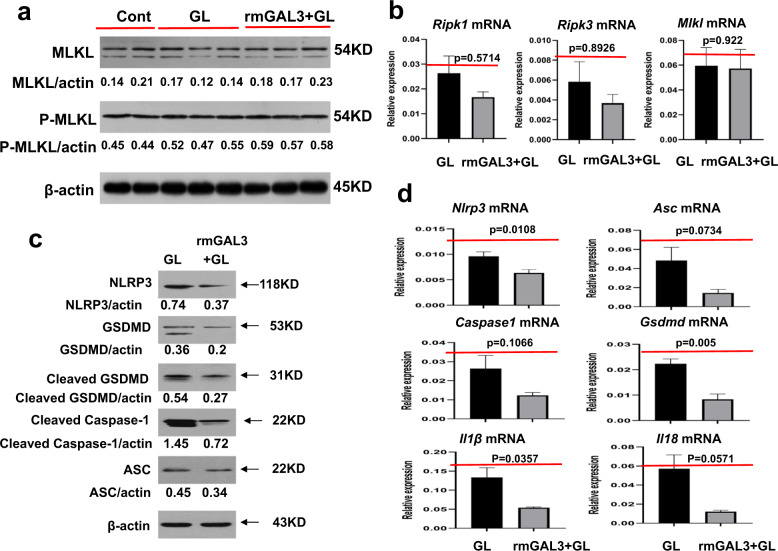
GAL3 exerts hepatoprotection in acutely injured mice by inhibiting pyroptosis but not necroptosis signalling. **a** The protein expression levels of necroptosis signalling molecules, including MLKL and p-MLKL, as detected by western blot in the livers of control and acutely injured mice with or without recombinant GAL3 treatment. **b** The mRNA expression levels of necroptosis signalling molecules, including *Ripk1*, *Ripk3* and *Mlkl*, in the livers of control and acutely injured mice with or without recombinant GAL3 treatment (*n* = 6–7). **c** The protein expression levels of pyroptosis signalling molecules, including NLRP3, GSDMD, cleaved GSDMD, ASC and cleaved caspase-1, as detected by western blot in the livers of control and acutely injured mice with or without recombinant GAL3 treatment. **d** The mRNA expression levels of pyroptosis signalling molecules, including *Nlrp3*, *Gsdmd*, *Asc*, *Caspase1*, *Il1β* and *Il18*, in the livers of control and acutely injured mice with or without recombinant GAL3 treatment (*n* = 4–6). Group comparisons were performed using Student’s *t* test or the Mann–Whitney *U* test.

Recently, we verified that M2-like macrophages exert hepatoprotection in ACLF by inhibiting necroptosis-S100A9-necroinflammation [14]. In view of the pivotal role of GAL3 in M2-like macrophage-mediated hepatoprotection, as mentioned above, we investigated whether the beneficial protective effects conferred by GAL3 can be ascribed to the inhibition of necroptosis signalling. Therefore, we analysed and compared the levels of necroptosis and ensuing necroinflammation in acutely injured mice with or without recombinant GAL3 pretreatment. Unexpectedly, the administration of recombinant GAL3 did not bring about any obvious differences in the expression of mixed-lineage kinase domain-like protein (MLKL) and p-MLKL, as detected by western blot (Fig. 6a). Consistent with these findings, the mRNA levels of *Ripk1*, *Ripk3* and *Mlkl* did not exhibit significant differences between these two groups (Fig. 6b). These findings suggest that the hepatoprotection conferred by GAL3 may not be explained by the inhibition of necroptosis signalling. Considering that the gene and protein expression levels of the NLR family pyrin domain containing 3 (NLRP3) were significantly reduced in response to the administration of recombinant GAL3, we speculated that another mode of programmed cell death probably holds an essential place in the protection conferred by GAL3 through interfering with NLRP3-related signalling. We then focused on pyroptosis, a novel form of proinflammatory programmed cell death, considering the importance of the NLRP3 inflammasome in the development of pyroptosis. To confirm the crucial role of pyroptosis in the hepatoprotection conferred by GAL3, we assessed and compared the levels of key molecules involved in pyroptosis signalling in acutely injured mice with or without recombinant GAL3 treatment. As a result, the protein expression levels of pyroptosis signalling molecules, including NLRP3, apoptotic speck-like protein with a caspase activating domain (ASC), and cleaved CASPASE1, detected by western blot analysis were obviously inhibited in galectin-3-treated mice upon insult. Importantly, gasdermin D (GSDMD) and cleaved GSDMD, critical effector molecules in pyroptosis signalling, exhibited downregulated expression in response to recombinant GAL3 (Fig. 6c). Consistently, the mRNA levels of *Nlrp3*, *Asc*, *Caspase1*, *Gsdmd*, *Il* (interleukin)*1β* and *Il18* were markedly downregulated in galectin-3-treated mice upon insult (Fig. 6d). Collectively, GAL3 exerts a protective effect against acute insult by inhibiting pyroptosis rather than necroptosis signalling.

### GAL3 inhibition abolishes the hepatoprotective effects conferred by GAL3 by promoting pyroptosis but not necroptosis signalling

**Fig. 7:**
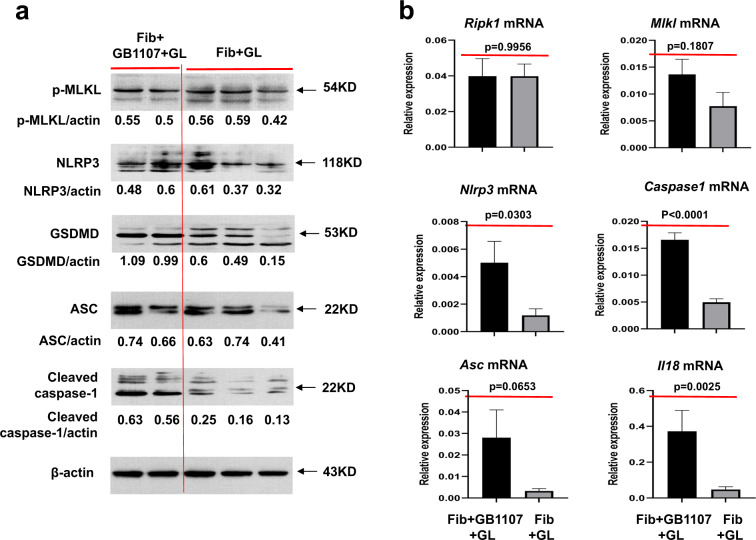
GAL3 inhibition abolishes the hepatoprotection in fibrotic mice against acute insult by upregulating pyroptosis rather than necroptosis signalling. **a** The protein expression levels of the necroptosis signalling molecule p-MLKL and pyroptosis signalling molecules, including NLRP3, GSDMD, ASC and caspase-1, as detected by western blot in the livers of fibrotic mice upon insult with or without GB1107 treatment. **b** The mRNA expression levels of necroptosis signalling molecules, including *Ripk1* and *Mlkl*, and pyroptosis signalling molecules, including *Nlrp3*, *Asc*, *Caspase1* and *Il18*, in the livers of fibrotic mice upon insult with or without GB1107 treatment (*n* = 5–8). Group comparisons were performed using Student’s *t* test or the Mann–Whitney *U* test.

Finally, we wanted to further verify the molecular mechanism underlying galectin-3-mediated hepatoprotection by inhibiting GAL3. To this end, we analysed and compared the levels of necroptosis signalling in fibrotic mice upon insult with or without the administration of the GAL3 inhibitor GB1107. Similarly, the inhibition of GAL3 did not result in significant changes in the gene levels of *Ripk1* and *Mlkl* or the protein expression of p-MLKL, as assessed by western blot in fibrotic mice upon insult (Fig. 7a, b). Thus, GAL3 did not confer hepatoprotection against acute insult in fibrotic mice by inhibiting necroptosis signalling. Therefore, we investigated the possible role of pyroptosis signalling in galectin-3-mediated hepatoprotection. According to our data, the protein expression levels of signalling molecules, including NLRP3, GSDMD, ASC and cleaved CASPASE1, were obviously enhanced in GB1107-treated fibrotic mice upon insult (Fig. 7a). In parallel, the mRNA levels of *Nlrp3*, *Caspase1*, *Asc* and *Il18* were obviously upregulated in fibrotic mice upon insult with the GAL3 inhibitor GB1107 (Fig. 7b). The above-mentioned results suggest that GAL3 carries out protective effects by inhibiting pyroptosis. Hence, GAL3 exhibits beneficial hepatoprotection by suppressing pyroptosis rather than necroptosis signalling.

## Discussion

Recently, we demonstrated for the first time that M2-like macrophages in the fibrotic liver exert beneficial hepatoprotection in ACLF by inhibiting necroptosis-S100A9-necroinflammation. In this work, we integrated GAL3 into this hepatoprotection and attempted to further dissect the molecular mechanism underlying M2-like macrophage-conferred hepatoprotection. We found the following: first, GAL3 is highly expressed in fibrotic livers; second, GAL3 exerts beneficial hepatoprotection against acute insult in control and fibrotic mice; third, GAL3 expression is closely correlated with M2-like macrophage activation, and M2-like macrophages seem to be the major cell source of GAL3; and fourth, GAL3 holds a crucial place in the hepatoprotection conferred by M2-like macrophages. Unexpectedly, we did not demonstrate that GAL3 exerts hepatoprotective effects by inhibiting necroptosis-S100A9-necroinflammation. As a result, our data showed that GAL3 exerts a protective effect against acute insult by inhibiting pyroptosis, another programmed death mode. Together, we documented that GAL3 promotes beneficial hepatoprotection conferred by M2-like macrophages in ACLF by inhibiting pyroptosis rather than necroptosis signalling. To the best of our knowledge, this is the first study to integrate GAL3 into the favourable hepatoprotection conferred by M2-like macrophages in ACLF. Importantly, we reported a novel mechanism by which GAL3 exerts favourable protection in ACLF, namely, GAL3 inhibits pyroptosis but not necroptosis signalling.

As described in the Introduction, the function of GAL3 in liver diseases is still a matter of debate. To elucidate the precise role of GAL3 in ACLF, we first compared the expression levels of GAL3 in control and fibrotic mice with or without acute challenge. Interestingly, GAL3 was obviously upregulated in the liver of fibrotic mice with or without acute insult but not in acutely injured mice, suggesting that GAL3 may be related to hepatic fibrosis. The colocalisation of GAL3 and SMA provided further support for the close correlation between GAL3 and fibrosis. Notably, the expression of GAL3 undermined along with the resolution of hepatic fibrosis, providing credible evidence for the intimate relationship between GAL3 and hepatic fibrosis. Our data are in accordance with the latest reports. For example, Nagasaki et al. confirmed that GAL3 promoted the activation of hepatic stellate cells and exacerbated fibrosis in nonalcoholic steatohepatitis [23]. Moreover, the number of SMA^+^GAL3^+^ cells was found to be increased with the severity of fibrosis in children with nonalcoholic fatty liver disease [24].

We previously demonstrated that injury resistance against acute insult occurs in the setting of hepatic fibrosis. To ascertain the function of GAL3 in hepatoprotection in the context of fibrosis, we performed a loss-of-function experiment focusing on GAL3. As expected, depleting GAL3 in fibrotic mice followed by acute insult aggravated hepatic damage, as shown by the markedly reduced survival rate and deteriorated histopathology. We also confirmed the hepatoprotective effects of GAL3 by adoptively transferring recombinant mouse GAL3 into control mice upon challenge. Sharply reduced ALT and AST levels as well as improved survival rate and hepatic histology were noted in mice receiving recombinant GAL3 compared with acutely injured mice. Therefore, GAL3 exerts a beneficial hepatoprotective effect in ACLF. This finding is consistent with a recent report by Zhao et al. [25], who found that *Lgals3* mRNA levels were lower in HBV-related ACLF patients than in chronic hepatitis B patients or healthy controls. Among ACLF patients, *Lgals3* mRNA levels were higher in survivors than in nonsurvivors.

Recently, we documented that M2-like macrophages assume a crucial protective role in our ACLF model. Next, we wondered whether the hepatoprotection mediated by GAL3 is correlated with M2-like macrophages. According to our data, GAL3 colocalised with CD206, a representative marker of M2-like macrophage activation. In particular, the mRNA levels of *Lgals3* exhibited positive correlations with those of M2 markers, including *Arg1*, *CD206*, *Ym1* and *Tgfβ*. Importantly, recombinant GAL3 pretreatment led to enhanced CD206 but much weaker iNOS in acutely injured mice. Collectively, the hepatoprotection mediated by GAL3 is closely correlated with M2-like macrophages. Our results partially coincide with the findings that GAL3 is essential for the production of M2-like or reparative macrophages [26, 27].

To verify the regulation between M2-like macrophages and GAL3, we conducted gain- and loss-of-function experiments with M2-like macrophages. According to our data, adoptive transfer of M2-like macrophages led to enhanced GAL3 expression, as assessed by immunostaining and quantitative PCR assays, in acutely injured mice. In contrast, depletion of M2-like macrophages resulted in significantly lowered expression of GAL3 in fibrotic mice upon insult. In vitro, *Lgals3* was highly expressed in M2-like macrophages compared with M0 or M1 macrophages. Clearly, M2-like macrophages act as a major cell source of GAL3. This finding is in accordance with the reports in several outstanding studies related to atherosclerosis [26], brain parasitic infection [28] or renal fibrosis [29].

We then explored whether GAL3 plays a pivotal role in the hepatoprotection conferred by M2-like macrophages. For this aim, we treated control mice with M2-like macrophages and GAL3 inhibitor simultaneously, followed by acute insult. As a result, the hepatoprotection conferred by M2-like macrophages vanished, as evidenced by markedly exaggerated histology in mice receiving a GAL3 inhibitor. However, when we transferred recombinant GAL3 into fibrotic mice upon M2-like macrophage depletion, followed by acute insult, the crippled hepatoprotection was restored, as shown by the obviously improved histology in mice receiving recombinant GAL3. Thus, GAL3 holds a crucial place in the hepatoprotection conferred by M2-like macrophages in ACLF.

Very recently, we reported that M2-like macrophages exert hepatoprotective effects in ACLF by inhibiting necroptosis-S100A9-necroinflammation [14]. On this basis, we attempted to confirm that GAL3 mediates the hepatoprotection conferred by M2-like macrophages by inhibiting necroptosis-S100A9-necroinflammation. Unexpectedly, our data from RT–qPCR and western blot analysis showed that recombinant GAL3 did not cause significant differences in the expression levels of necroptosis signalling molecules, including RIPK1, RIPK3, MLKL and/or P-MLKL. Interestingly, the expression of NLRP3 was markedly suppressed in acutely injured mice with GAL3 pretreatment. Thus, we speculated that another cell death mode related to NLRP3 may be involved in the protective effect mediated by GAL3. In view of the critical role of NLRP3 in pyroptosis, we assumed that GAL3 may exert hepatic protection by inhibiting pyroptosis signalling. Pyroptosis is a proinflammatory programmed cell death. It can be driven by a wide array of extracellular stimuli, including PAMPs and DAMPs. In the canonical model, NLRP3 responds to these stimuli and is activated indirectly through potassium efflux. Then, NLRP3 oligomerizes and subsequently activates caspase-1 via the adaptor protein ASC. Caspase-1 processes and activates IL1β and IL18, while it cleaves GSDMD to release the membrane pore-forming GSDMD-N domain. The GSDMD-N domain oligomerizes and forms pores in the cell membrane, leading to cell lysis and IL1β/IL18 release [30–33].

To test our hypothesis that GAL3 may exert hepatic protection by inhibiting pyroptosis signalling, we compared the expression levels of pyroptosis signalling molecules, including NLRP3, ASC, cleaved CASPASE1, GSDMD, cleaved GSDMD, IL1β and IL18, in the livers of acutely injured mice with or without recombinant GAL3 treatment. Western blot and real-time PCR analyses showed that the administration of recombinant GAL3 inhibited the intensity of pyroptosis signalling. We also validated this hypothesis from the perspective of GAL3 depletion. GAL3 inhibition did not bring about significant changes in the necroptosis signalling molecules. Instead, the mRNA and protein levels of pyroptosis signalling molecules were markedly increased in the livers from fibrotic mice upon insult with GB1107 treatment. Therefore, GAL3 exerts protective effects against acute insult in ACLF by inhibiting pyroptosis signalling.

**Fig. 8:**
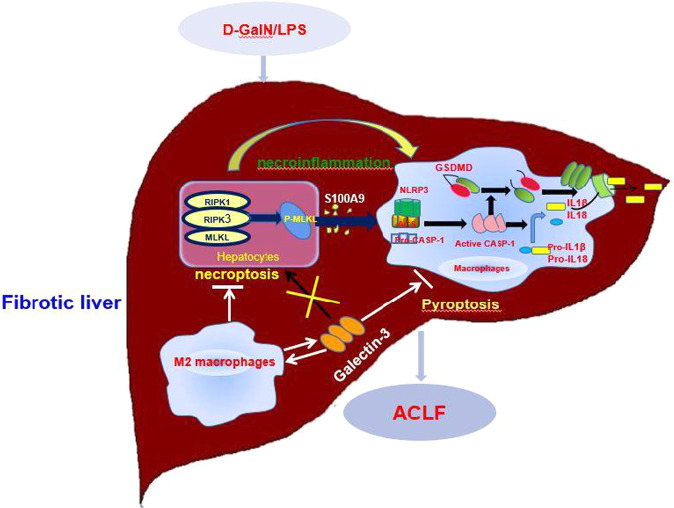
An illustration of the pivotal role of GAL3 in the hepatoprotection conferred by M2-like macrophages in ACLF. GAL3 is produced by M2-like macrophages and promotes M2-like macrophage activation, then exerts the hepatoprotective effects in ACLF through inhibiting pyroptosis but not necroptosis signalling.

In summary, we confirmed that GAL3 plays a pivotal role in the hepatoprotection conferred by M2-like macrophages in ACLF by inhibiting pyroptosis but not necroptosis signalling (Fig. 8). Our findings provide novel insights into the pathogenesis of ACLF, which makes it possible to treat ACLF patients by modulating GAL3.

## Materials and methods

Details are provided in the Supplementary Materials.

### Animals

Six-week-old male BALB/c mice were used for macrophage isolation and the development of acute and chronic hepatic injury models. All experiments were approved by the Institutional Animal Care and Use Committee of Beijing YouAn Hospital, Capital Medical University.

### Animal protocol

The experimental protocol was as follows: (1) Control: BALB/c mice were given mineral oil, PBS or DMSO as appropriate. (2) Induction of hepatic fibrosis: BALB/c mice were injected intraperitoneally with 20% CCl_4_ (2 μl/g, in mineral oil) twice a week for 6 weeks. (3) Pharmacological interventions: Control and fibrotic mice were intragastrically administered a special inhibitor targeting GAL3, namely, GB1107 (10 mg/kg) or recombinant GAL3 (6 μg/mouse) by tail vein injection. (4) Acute insult: Mice were challenged intraperitoneally with D-GalN (500 μg/g, Sigma) plus LPS (10 ng/g, Invitrogen). Sera and liver tissues were harvested 24 h after acute challenge for analysis.

### Evaluation of liver injury

Hepatic damage was evaluated by serum ALT and AST levels and hepatic histology. Histological severity was scored blindly by experienced pathologists [34].

### Statistical analysis

The results are expressed as the mean ± standard error of the mean or median (min, max). Group comparisons were performed using Student’s *t* test, Mann–Whitney *U* test, and one-way ANOVA followed by Tukey’s multiple comparison test, as appropriate. Survival analysis was conducted with the Kaplan–Meier method, and the log-rank test was used to compare the differences in survival between treatment groups. Statistics and graphs were generated using Prism 6.0 software (GraphPad Software Inc., San Diego, CA, USA). *P* < 0.05 was considered statistically significant.

## Supplementary information

supporting materials final

WB RAW DATA

aj-checklist

mail about the author list

AJE certificate

## Data Availability

All data generated during this study are included in this published article and its Supplementary Information.
