# Supplementary material for: Galectin-3 critically mediates the hepatoprotection conferred by M2-like macrophages in ACLF by inhibiting pyroptosis but not necroptosis signalling
Source: Cell Death Dis. 2022 Sep 8;13(9):775. doi: 10.1038/s41419-022-05181-1 (PMC9458748; doi:10.1038/s41419-022-05181-1)
Supplement: Supplementary file 1 — supporting materials final [file 41419_2022_5181_MOESM1_ESM.docx]

**Galectin-3 critically mediates the hepatoprotection conferred by M2-like macrophages in ACLF by inhibiting pyroptosis but not necroptosis signalling**

Li Bai^1, 2^, Wang Lu^1, 2^, Shan Tang^3^, Huixin Tang^1, 2^, Manman Xu^1, 2^, Chen Liang^3^, Sujun Zheng^3^, Shuang Liu^1, 2^, Ming Kong^1, 2^, Zhongping Duan^1, 2^, Yu Chen^1, 2†^

**Abbreviations:** SPF, specific pathogen free; GAL3, galectin-3; ALT, alanine aminotransferase; AST, aspartate aminotransferase; H&E, hematoxylin-eosin; qPCR, quantitative polymerase chain reaction; IF, immunofluorescence; IHC, immunohistochemistry; PVDF, polyvinylidene difluoride; PBS, phosphate-buffered saline; DMSO, dimethylsulfoxide; CCl_4_, carbon tetrachloride; MLKL, mixed-lineage kinase domain-like protein; NLRP3, NLR family pyrin domain containing 3; GSDMD, gasdermin D; ASC, apoptotic speck-like protein with a caspaseactivating domain; IL, interleukin; D-GalN, D-galactosamine; LPS, lipopolysaccharide;

**Supporting Materials**

**Animals**

Six-week-old male BALB/c mice were purchased from the Laboratory Animal Breeding Center of Beijing LongAn, Beijing, China. Mice were maintained in a specific pathogen-free (SPF) environment at 22 °C-24 °C with a 12-hour light-dark cycle. Animals were fed standard laboratory chow and given free access to water. The Guide for the Care and Use of Laboratory Animals was followed during animal care and experimental procedures. The animal protocol was approved by the Institutional Animal Care and Use Committee of Beijing YouAn Hospital, Capital Medical University.

**Animal protocol**

The experimental protocol was as follows: (1) Control: BALB/c mice were given mineral oil, phosphate-buffered saline (PBS) or dimethylsulfoxide (DMSO) as appropriate. (2) The induction of hepatic fibrosis: BALB/c mice were injected intraperitoneally with 20% carbon tetrachloride (CCl_4_, 2 μl/g, in mineral oil) twice a week for 6 weeks. (3) [Pharmacological](https://cn.bing.com/dict/search?q=Pharmacological&FORM=BDVSP6&mkt=zh-cn) [interventions](https://cn.bing.com/dict/search?q=Interventions&FORM=BDVSP6&mkt=zh-cn): Control and fibrotic mice were intragastrically administered a special inhibitor targeting GAL3, namely, GB1107 (10 mg/kg, MedChemExpress) or recombinant GAL3 (6 μg/mouse, R&D) by tail vein injection. (4) Acute insult: Mice were challenged intraperitoneally with D-galactosamine (D-GalN, 500 μg/g, Sigma) plus lipopolysaccharide (LPS, 10 ng/g, Invivogen). Sera and liver tissues were harvested 24 hours after acute challenge for analysis.

**Evaluation of liver injury**

The levels of alanine aminotransferase (ALT) and aspartate aminotransferase (AST) in serum were measured using an automatic chemical analyser (Hitachi 7600, Japan) according to an automated procedure.

Formalin-fixed and paraffin-embedded liver tissues were sectioned (3 μm) and stained with haematoxylin-eosin (H&E) according to a standard protocol. Histological images were captured using an Olympus Bx51 microscope (Olympus America, Melville, NY, USA) and processed with cellSens imaging software (version 1.4.1). The histological severity of hepatic damage was assessed and scored blindly by experienced pathologists [1].

**Reverse transcription and SYBR Green quantitative polymerase chain reaction (qPCR)**

Total RNA from liver tissues and cultured cells was extracted using TRIzol reagent (Thermo Fisher Scientific, Waltham, MA, USA) following the manufacturer’s instructions. Then, 1 μg RNA was reverse transcribed into cDNA using the AMV retrotranscriptase system (TaKaRa, Dalian, Liaoning, China). qPCR amplifications were run in triplicate on an ABI StepOne Plus System (Thermo Fisher Scientific) using SYBR Green reaction mix (TaKaRa). In a 20 μl reaction volume, the following reagents were added: 1× SYBR Green PCR master mix, template cDNA, 0.5 mM each primer, and ROX. The thermal cycling protocol was 95 °C for 10 min, followed by 40 cycles of 95 °C for 15 s and 60 °C for 1 min. The primers used in this work were designed by Primer 3 (Version 0.4.0) and are listed in Supplementary Table 1. The relative expression of the target gene was calculated and normalized to the expression of the reference gene *Gapdh*.

**Immunofluorescence analysis**

Immunofluorescent staining was performed on frozen liver sections (5 μm), as previously described [2]. Liver sections were stained with the following primary antibodies: anti-mouse GAL3 (1:300; Abcam, Cambridge, MA, USA), FITC Mouse Anti-iNOS/NOS Type II (1:200; BD Transduction Laboratories™, San Jose, CA, USA), PE anti-mouse CD206 (MMR) (1:200; BioLegend Inc., San Diego, CA, USA), and anti-actin, α-smooth muscle (1:300; Sigma, St Louis, MO, USA). For indirect immunofluorescent staining of GAL3, liver sections were incubated with PE- or FITC-conjugated donkey anti-rabbit IgG (1:500). The images were captured with a Nikon ECLIPSE Ti inverted fluorescence microscope and processed with NIS-Elements F 3.0 Software (Nikon Corporation, Tokyo, Japan).

**Immunohistochemistry (IHC) analysis**

Liver sections were deparaffinized in xylene and rehydrated through descending grades of ethanol to deionized water. Then, the slides were treated with 5% H_2_O_2_ for 15 min followed by antigen retrieval for 3 min in EDTA buffer using a pressure cooker. Nonspecific proteins were blocked with goat serum for 20 min at 37 °C. Then, the sections were incubated with anti-GAL3 antibodies (1:50, Abcam) overnight at 4 °C. After washing three times with PBS, the sections were incubated with goat anti-rabbit secondary antibodies (Zhongshan Golden Bridge Biotechnology Co., Ltd., Beijing, China) for 20 min at 37 °C. After washing with PBS, the sections were incubated with horseradish peroxidase-conjugated tertiary antibodies for 20 min at 37 °C. After that, the slides were incubated with diaminobenzidine and counterstained with haematoxylin, followed by dehydration and stabilization with mounting medium. Images were captured using an Olympus Bx51 microscope and processed with cellSens standard software. The expression of target proteins was quantified by Image J software.

**Western blot analysis**

Whole proteins from frozen liver tissues or cells were extracted using RIPA lysis buffer supplemented with Halt™ protease inhibitor cocktail (Thermo Fisher Scientific). Samples were then subjected to 10% SDS–PAGE and transferred onto polyvinylidene difluoride (PVDF) membranes (Thermo Fisher Scientific). The membranes were blocked with 5% BSA for 1 h at room temperature and then probed with the following primary antibodies overnight at 4 °C: RIP (D94C12) XP rabbit mAb (1:1000; Cell Signaling Technology), anti-MLKL antibody (1:1000, Abcam), phospho-MLKL (Ser345)(D6E3G) rabbit mAb (1:1000; CST), anti-NLRP3 antibody (1:1000; Abcam), ASC/TMS1 (D2 W8U) rabbit mAb (1:1000; CST), cleaved caspase-1 (Asp296) (E2G2I) rabbit mAb (1:1000; CST), gasdermin D (L60) (1:1000; CST), cleaved gasdermin D (Asp276) (E3E3P) rabbit mAb (1:1000; CST), and β-actin. After washing, the membranes were incubated with HRP-conjugated anti-rabbit IgG for 1 h at room temperature. The protein bands were visualized by Luminol ECL reagent (Thermo Fisher Scientific). The expression of target proteins was normalized to that of β-actin.

**Statistical analysis**

The results are expressed as the mean ± standard error of the mean or median (Min, Max). Group comparisons were performed using Student’s *t* test, the Mann–Whitney U test, and one-way ANOVA followed by Tukey’s multiple comparison test, as appropriate. Survival analysis was conducted with the Kaplan–Meier method, and the log-rank test was used to compare the differences in survival between treatment groups. Statistics and graphs were generated using Prism 6.0 software (GraphPad Software Inc., San Diego, CA, USA). *P*<0.05 was considered statistically significant.

**REFERENCES**

1. Wang F, Xue Y, Yang J, Lin F, Sun Y, Li T, et al. Hepatoprotective effect of apple polyphenols against concanavalin A-induced immunological liver injury in mice. Chem Biol Interact. 2016;258:159-65.

2. Bai L, Kong M, Zheng Q, Zhang X, Liu X, Zu K, et al. Inhibition of the translocation and extracellular release of high-mobility group box 1 alleviates liver damage in fibrotic mice in response to D-galactosamine/lipopolysaccharide challenge. Mol Med Rep. 2016;13:3835-41.

**Supplementary Table**

**Supplementary Table 1. The primers used in this work**

| **Gene** | **Sense** | **Anti-sense** | **Accession** | **Locations** | **Concentrations (Final)** | **Amplification Efficiency（%）** |
| --- | --- | --- | --- | --- | --- | --- |
| *Gapdh* | 5’- aac ttt ggc att gtg gaa gg -3’ | 5’- aca cat tgg ggg tag gaa ca -3’ | NM_001289726 | 585-807 | 250 nM | 95.9 |
| *Lgals3* | 5’- cag tgc tcc tgg agg cta tc -3’ | 5’- att gaa gcg ggg gtt aaa gt -3’ | NM_001145953 | 451-658 | 250 nM | 97.7 |
| *Ripk1* | 5’- ctg ggc ttc act gag tct ca -3’ | 5’- tca agt ggt tca gca ggt ct -3’ | NM_001359997 | 2168-2351 | 250 nM | 102.29 |
| *Ripk3* | 5’- gta ctt gga ccc aga gct gt -3’ | 5’- ctg tca cac act gtt tcc cg -3’ | NM_001164107 | 771-926 | 250 nM | 102.95 |
| *Mlkl* | 5’- aag aag aac ctg ccc gat ga -3’ | 5’- ctg gct gac atc tga aac gg -3’ | NM_001310613 | 532-768 | 250 nM | 99.88 |
| *Il1β* | 5’- gcc cat cct ctg tga ctc at -3’ | 5’- agg cca cag gta ttt tgt cg-3’ | NM_008361 | 387-616 | 250 nM | 98.63 |
| *Il18* | 5'- caa agt gcc agt gaa ccc cag ac -3' | 5'- aca gag agg gtc aca gcc agt c -3' | NM_001357221 | 655-740 | 250 nM | 108.08 |
| *Asc* | 5'- gga cgg agt gct gga tgc ttt g -3' | 5'- cat ctt gtc ttg gct ggt ggt ctc -3' | NM_023258 | 595-685 | 250 nM | 101.43 |
| *Caspase1* | 5'- ata caa cca ctc gta cac gtc ttg c -3' | 5'- tcc tcc agc agc aac ttc att tct c -3' | NM_009807 | 667-781 | 250 nM | 104.31 |
| *Gsdmd* | 5'- cga tgg gaa cat tca ggg cag ag -3' | 5'- aca cat tca tgg agg cac tgg aac -3' | NM_026960 | 408-511 | 250 nM | 99.35 |
| *Tgfβ* | 5’- ttg ctt cag ctc cac aga ga - 3’ | 5’- tgg ttg tag agg gca agg ac -3’ | NM_011577 | 1719-1901 | 250 nM | 98.81 |
| *Nos2* | 5'- ggt ctt tga aat ccc tcc tga -3' | 5'- agc tcc tgg aac cac tcg ta -3' | NM_001313921 | 939-1013 | 250 nM | 99.26 |
| *Arg1* | 5’- ctg gca gtt gga agc atc tct -3’ | 5’- gtg agc atc cac cca aat gac -3’ | NM_007482 | 409-480 | 250 nM | 96.97 |
| *CD206* | 5’- atg cca agt ggg aaa atc tg -3’ | 5’- tgt agc agt ggc ctg cat ag -3’ | NM_008625 | 1070-1222 | 250 nM | 102.33 |
| *Ym1* | 5’- atc tat gcc ttt gct gga atg c -3’ | 5’- tga atg aat atc tga cgg ttc tga g -3’ | NM_009892 | 206-405 | 250 nM | 101.36 |
| *Nlrp3* | 5’- atg ctg ctt cga cat ctc ct -3’ | 5’- aac caa tgc gag atc ctg ac -3’ | NM_001359638 | 2484-2679 | 250 nM | 99.39 |
| *CD86* | 5’- cac gag ctt tga cag gaa ca -3’ | 5’- tta ggt ttc ggg tga cct tg -3’ | NM_019388 | 371-616 | 250 nM | 101.30 |
