# Supplementary material for: Galectin-3 critically mediates the hepatoprotection conferred by M2-like macrophages in ACLF by inhibiting pyroptosis but not necroptosis signalling
Source: Cell Death Dis. 2022 Sep 8;13(9):775. doi: 10.1038/s41419-022-05181-1 (PMC9458748; doi:10.1038/s41419-022-05181-1)

Figure 6a

P-MLKL

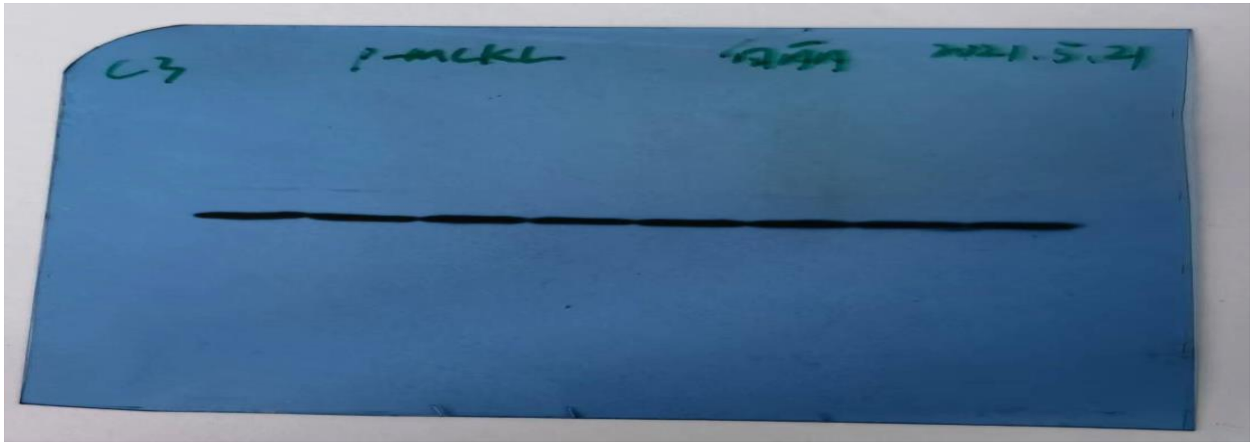

MLKL

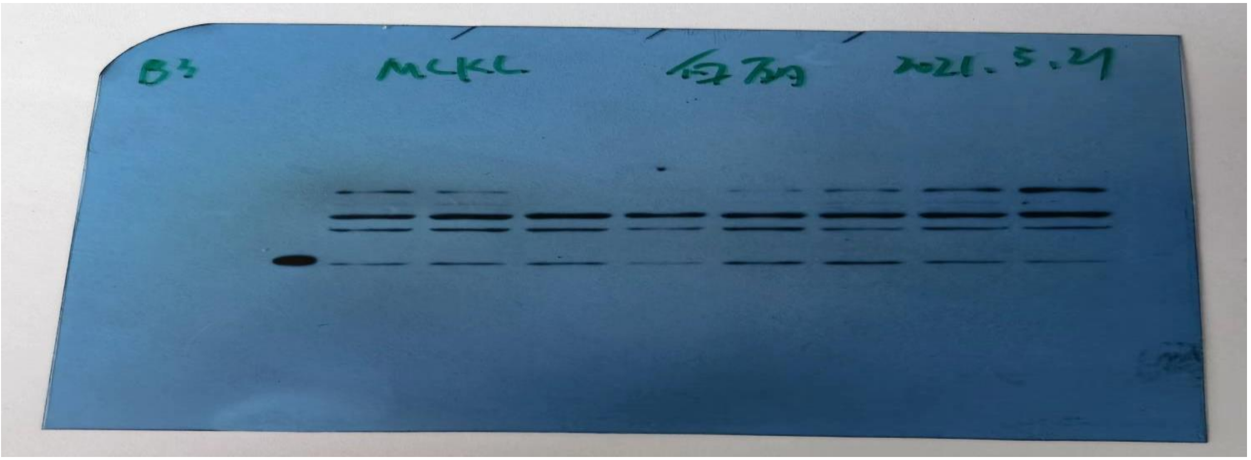

ACTIN

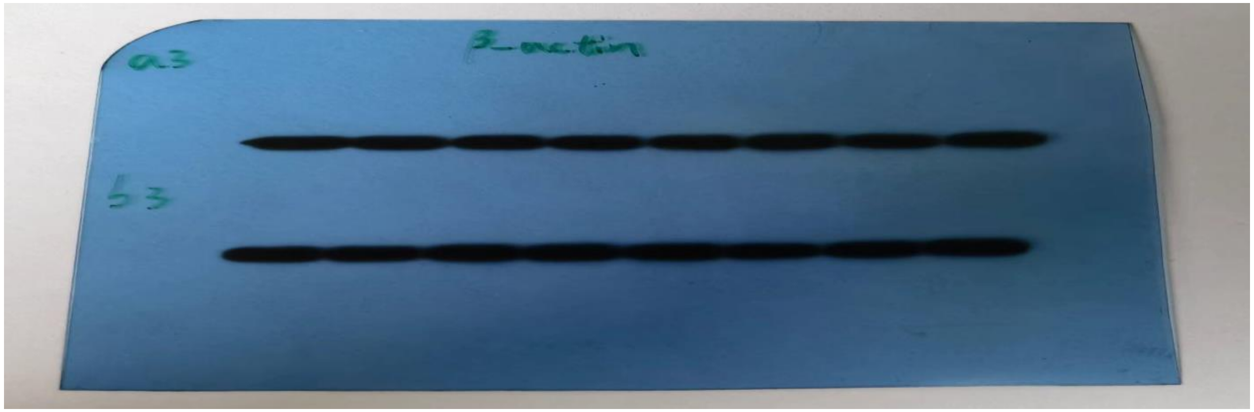

Figure 6c

GSDMD

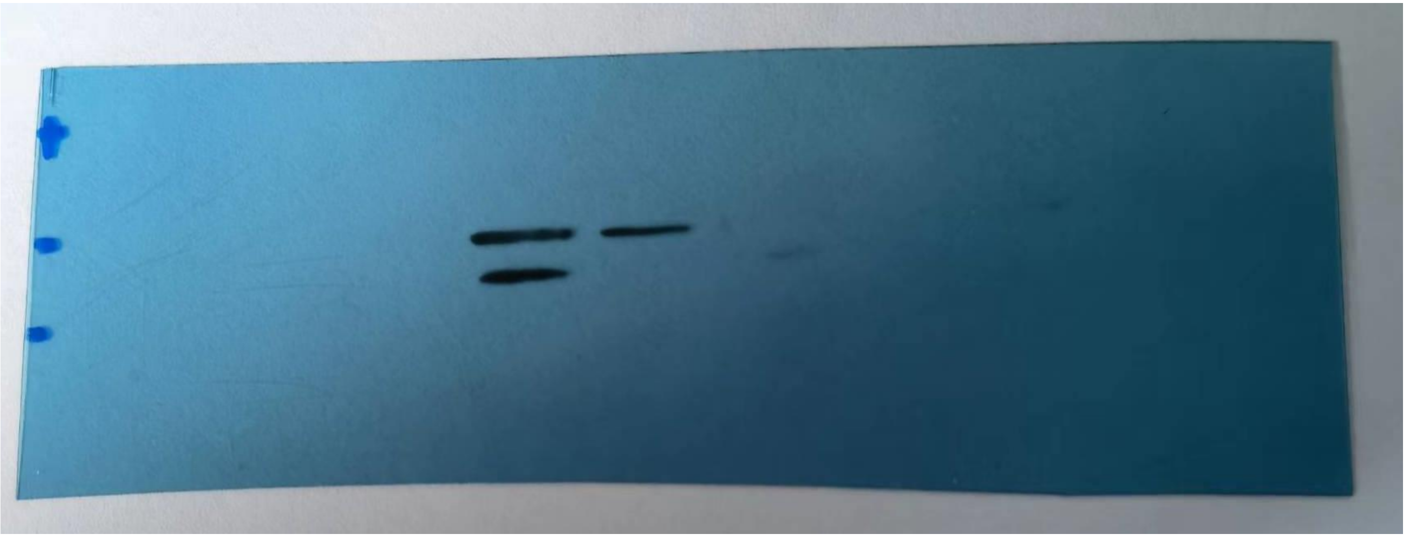

NLRP3

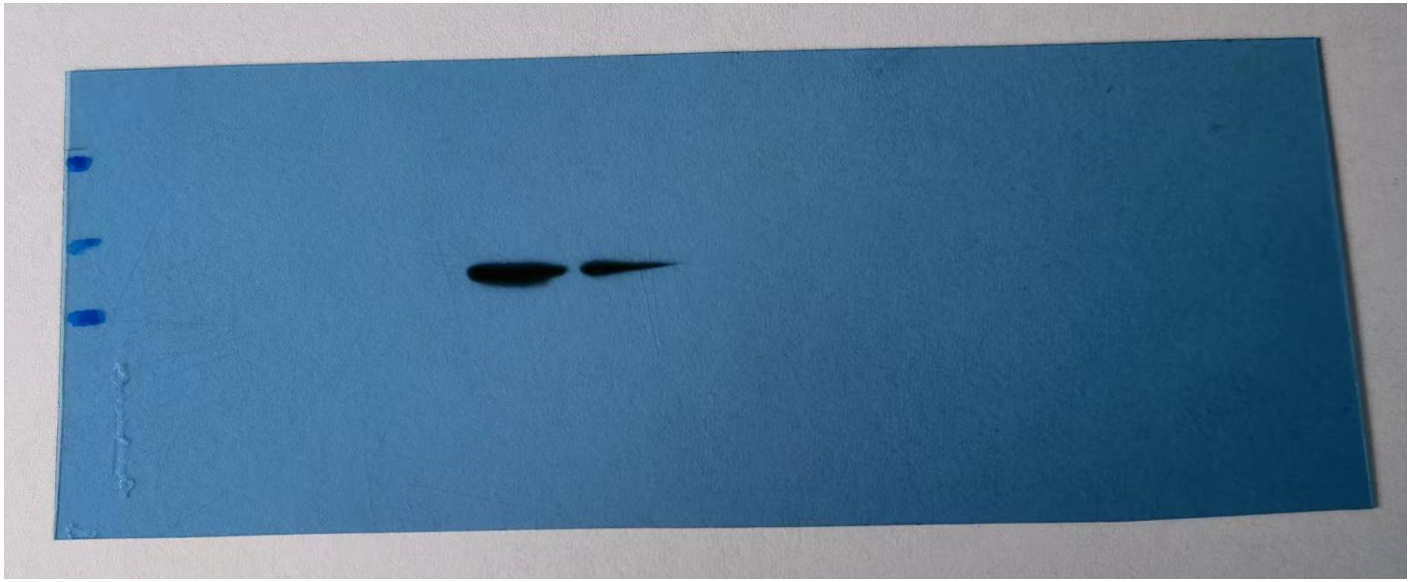

Figure 6c

Cleaved caspase-1

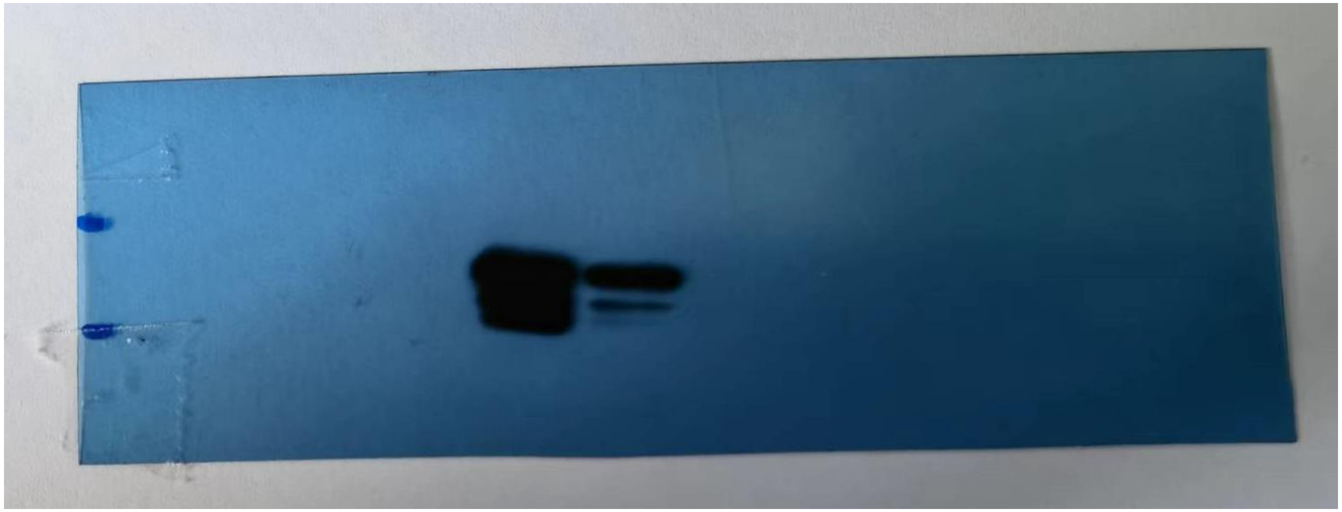

Cleaved GSDMD

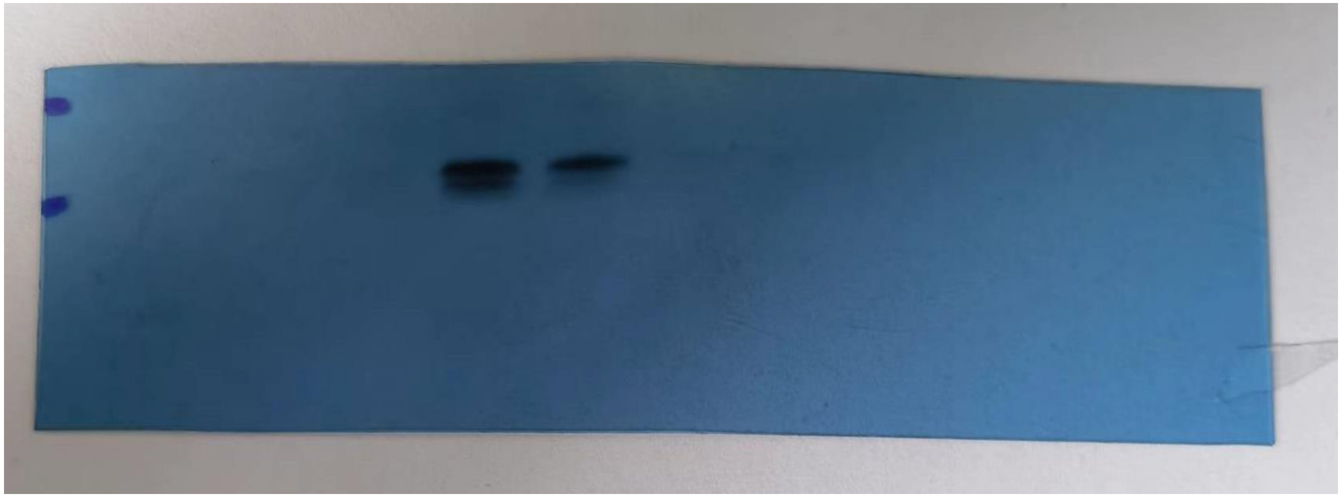

Figure 6c

ACTIN

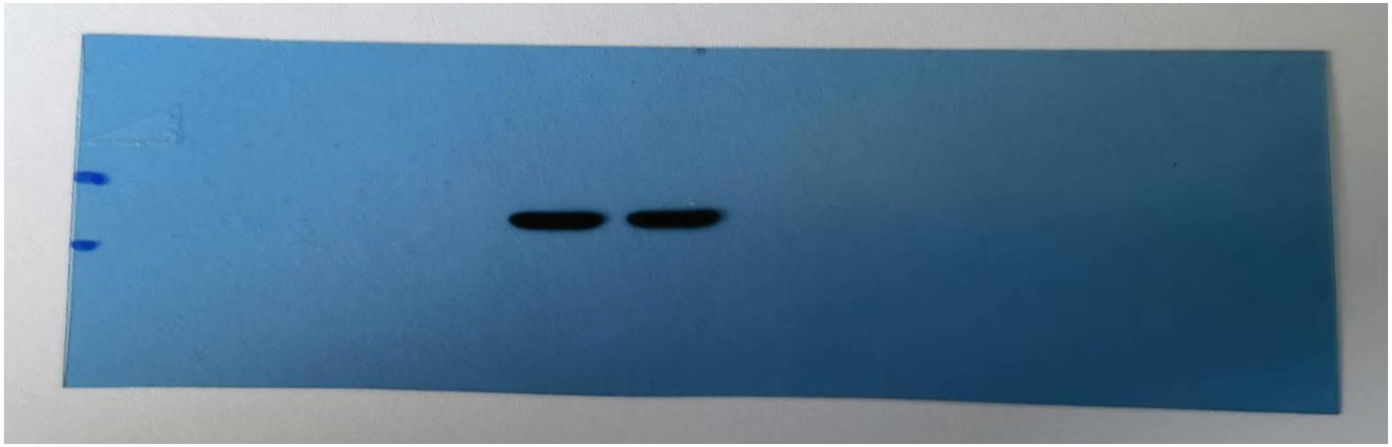

ASC

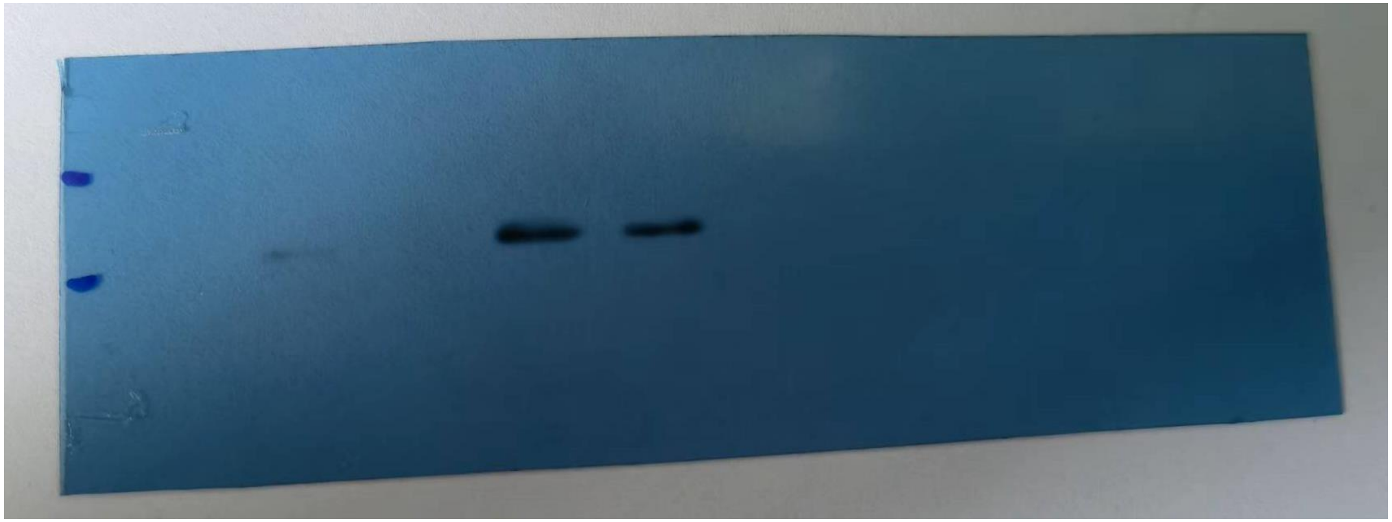

Figure 7a

P-MLKL

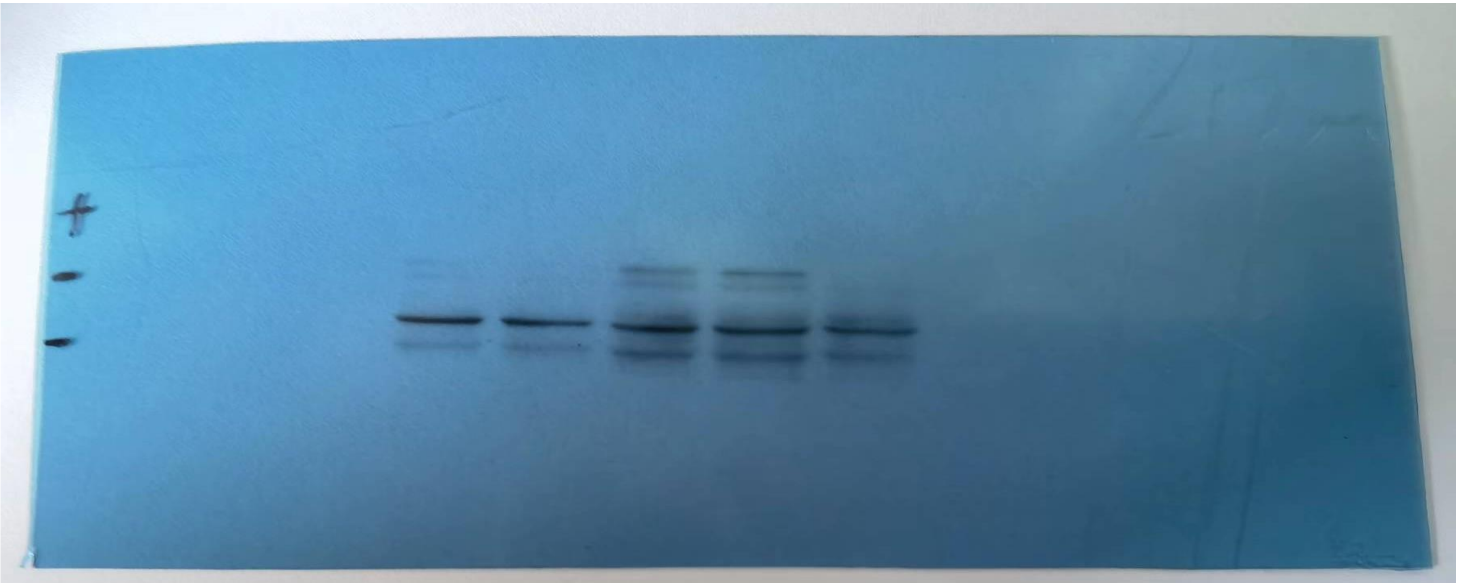

NLRP3

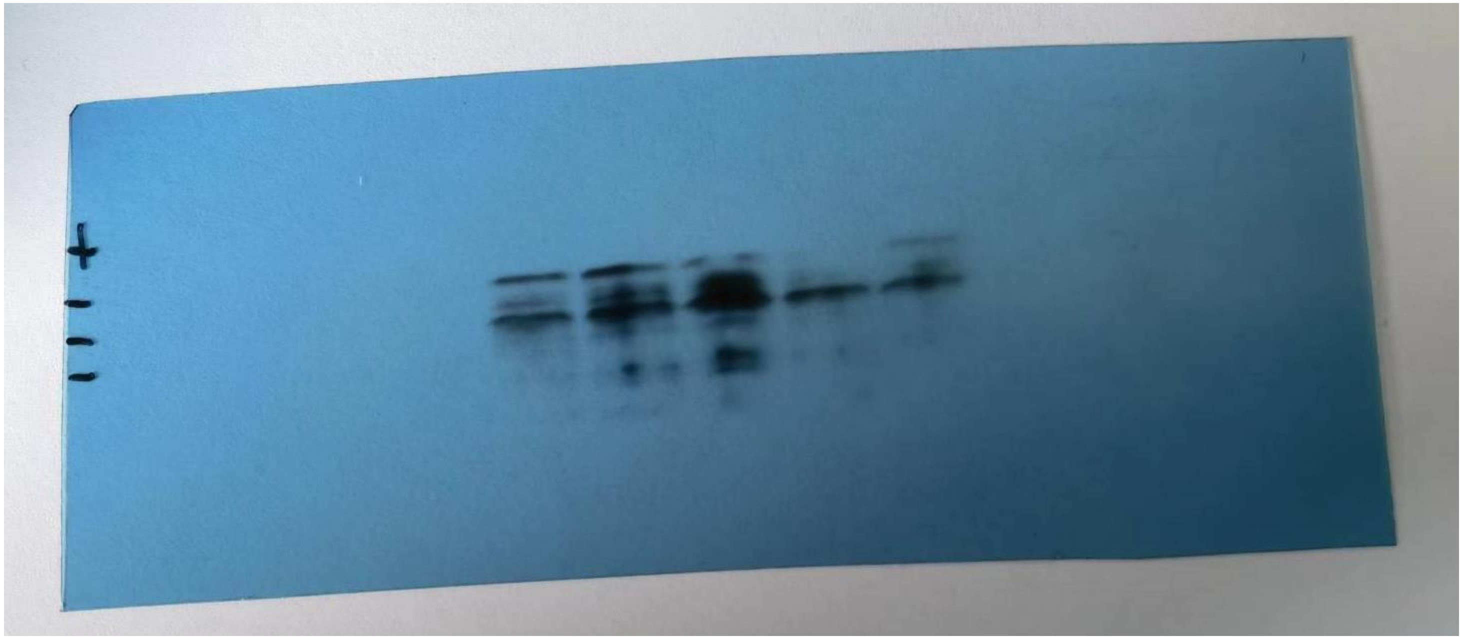

Figure 7a

GSDMD

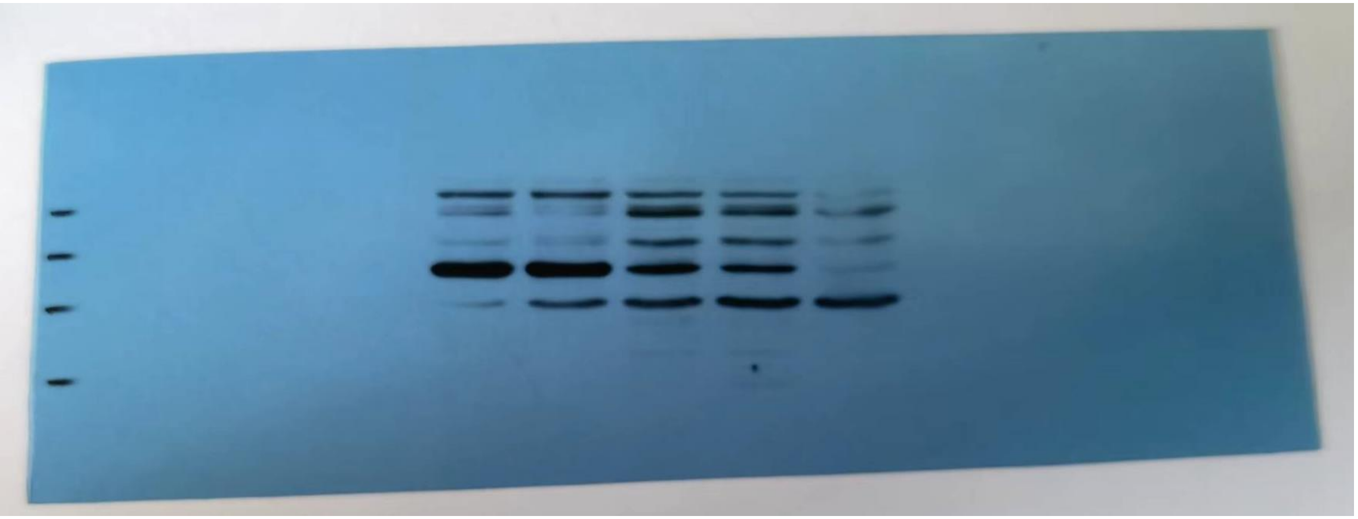

ASC

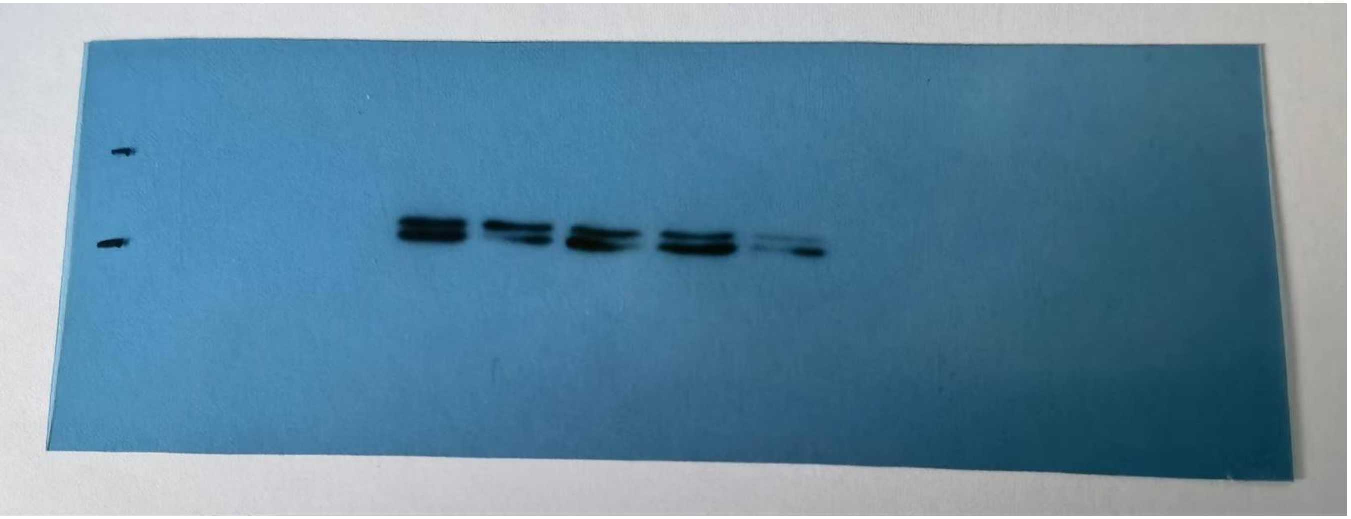

Figure 7a

B-ACTIN

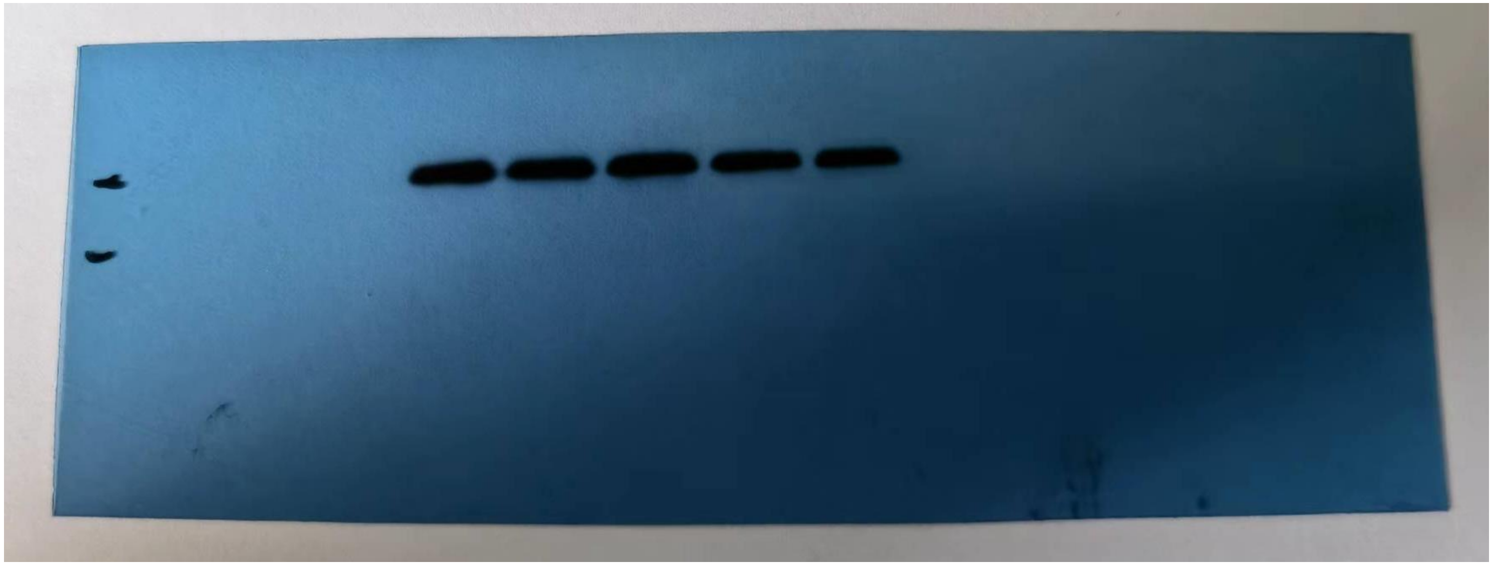

Cleaved caspase-1

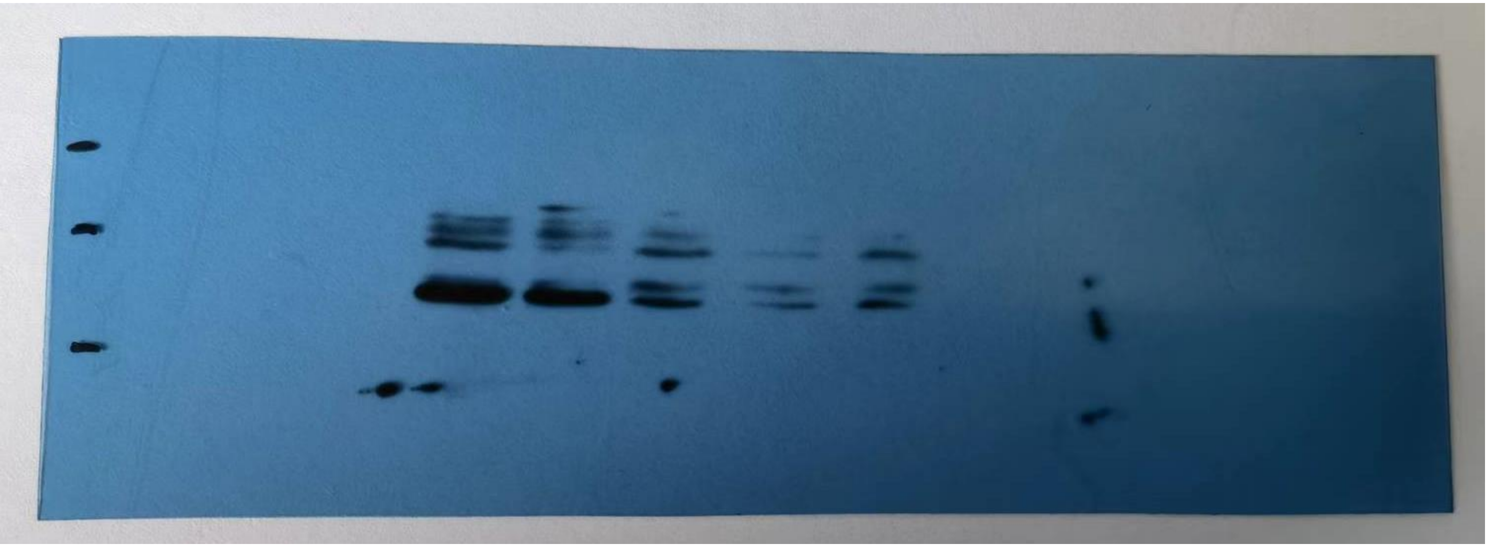

Supplement: Supplementary file 2 — WB RAW DATA [file 41419_2022_5181_MOESM2_ESM.pdf]
