# Supplementary material for: Galectin-3 critically mediates the hepatoprotection conferred by M2-like macrophages in ACLF by inhibiting pyroptosis but not necroptosis signalling
Source: Cell Death Dis. 2022 Sep 8;13(9):775. doi: 10.1038/s41419-022-05181-1 (PMC9458748; doi:10.1038/s41419-022-05181-1)
Supplement: Supplementary file 4 — mail about the author list [file 41419_2022_5181_MOESM4_ESM.pdf]

# E-mail about the author list

about the author list

优化阅读 | 精简信息

发件人: chenyurgg<chenyurgg@163.com>

收件人: tender78<tender78@126.com> duan2517abc<duan2517abc@126.com> zhengsujun73<zhengsujun73@163.com> shuangliu186<shuangliu186@126.com> kongmingrgg<kongmingrgg@163.com>

还有5个联系人 保存所有收件人

时 间: 2022年08月06日 16:38 (星期六)

发送状态: 发送成功 查看详情

翻译成中文

租银行保险柜2022年多少钱? 立即咨询 广告

Hi, everyone

In the work related to galectin-3, Manman Xu and Huixin Tang supplemented the experiments according to the reviews' suggestion. So, I suggest to add Manman Xu and Huixin Tang to the list of the authors. And the new list of authors is as follows: Li Bai, Wang Lu, Shan Tang, Huixin Tang, Manman Xu, Chen Liang, Sujun Zheng, Shuang Liu, Ming Kong, Zhongping Duan, Yu Chen. Please let me know if you agree with this list.

Yu Chen

Re:about the author list

优化阅读 | 精简信息

发件人: Zhongping Duan<duan2517abc@126.com>

收件人: chenyurgg<chenyurgg@163.com>

时 间: 2022年08月06日 16:42 (星期六)

翻译成中文

【升级】邮箱会员，更安全的邮箱体验，更大的存储空间，提高办公效率! 广告

I agree with the new author list.
Zhongping Duan

At 2022-08-06 16:38:23, "chenyurgg" <chenyurgg@163.com> wrote:

- 隐藏引用文字 -

Hi, everyone

In the work related to galectin-3, Manman Xu and Huixin Tang supplemented the experiments according to the reviews' suggestion. So, I suggest to add Manman Xu and Huixin Tang to the list of the authors. And the new list of authors is as follows: Li Bai, Wang Lu, Shan Tang, Huixin Tang, Manman Xu, Chen Liang, Sujun Zheng, Shuang Liu, Ming Kong, Zhongping Duan, Yu Chen. Please let me know if you agree with this list.

Re:about the author list

优化阅读 | 精简信息

发件人: tender78<tender78@126.com>

收件人: chenyurgg<chenyurgg@163.com>

时 间: 2022年08月06日 16:41 (星期六)

翻译成中文

租银行保险柜2022年多少钱? 立即咨询 广告

I agree with the new author list.
Li Bai

--

Li Bai,Ph.D.

Artificial Liver Treatment & Trainig Center
Beijing Youan Hospital
Capital Medical University
Beijing,100069,China

Re:about the author list

发件人： zhengsujun73<zhengsujun73@163.com>

收件人： chenyurgg<chenyurgg@163.com>

时　间： 2022年08月06日 16:47 (星期六)

优化阅读 | 精简信息

翻译成中文

贵重物品怎么放?银行授权网点,24h存取 立即咨询 广告

I agree with that.
Sujun Zheng

At 2022-08-06 16:38:23, "chenyurgg" <chenyurgg@163.com> wrote:

- 隐藏引用文字 -

> Hi，everyone
>
> In the work related to galectin-3, Manman Xu and Huixin Tang supplemented the experiments according to the reviews' suggestion. So, I suggest to add Manman Xu and Huixin Tang to the list of the authors. And the new list of authors is as follows: Li Bai, Wang Lu, Shan Tang, Huixin Tang, Manman Xu, Chen Liang, Sujun Zheng, Shuang Liu, Ming Kong, Zhongping Duan, Yu Chen. Please let me know if you agree with this list.

Re: about the author list

发件人： shuangliu186@126.com<shuangliu186@126.com>

收件人： chenyurgg<chenyurgg@163.com>

时　间： 2022年08月06日 16:49 (星期六)

精简信息

翻译成中文

贵重物品怎么放?银行授权网点,24h存取 立即咨询 广告

I agree with the new author list.
Shuang Liu

---- Replied Message ----

From	chenyurgg<chenyurgg@163.com>
Date	08/06/2022 16:38
To	tender78@126.com<tender78@126.com>、duan2517abc@126.com<duan2517abc@126.com>、zhengsujun73@163.com<zhengsujun73@163.com>、shuangliu186@126.com<shuangliu186@126.com>、kongmingrgg@163.com<kongmingrgg@163.com>、liangchenrgg@163.com<liangchenrgg@163.com>、tangshanrgg@163.com<tangshanrgg@163.com>、luwangrgg@163.com<luwangrgg@163.com>、tanghuixinrgg@163.com<tanghuixinrgg@163.com>、xumanmanrgg@163.com<xumanmanrgg@163.com>
Subject	about the author list

Re: about the author list

发件人： kongmingrgg@163.com<kongmingrgg@163.com>

收件人： chenyurgg<chenyurgg@163.com>

时　间： 2022年08月06日 16:50 (星期六)

精简信息

翻译成中文

2022北京租一个银行保管箱，要多少钱? 立即咨询 广告

I agree with the new author list.
Ming Kong

Re: about the author list

发件人： liangchenrgg@163.com<liangchenrgg@163.com>

收件人： chenyurgg<chenyurgg@163.com>

时　间： 2022年08月06日 16:51 (星期六)

精简信息

翻译成中文

贵重物品怎么放?银行授权网点,24h存取 立即咨询 广告

I agree with the new author list.
Chen Liang

Re: about the author list

精简信息

发件人: tangshanrgg@163.com<tangshanrgg@163.com>

收件人: chenyurgg<chenyurgg@163.com>

时 间: 2022年08月06日 16:52 (星期六)

翻译成中文

【升级】邮箱会员，更安全的邮箱体验，更大的存储空间，提高办公效率！ 广告

I agree with the new author list.
Shan tang

Re: about the author list

精简信息

发件人: luwangrgg@163.com<luwangrgg@163.com>

收件人: chenyurgg<chenyurgg@163.com>

时 间: 2022年08月06日 16:52 (星期六)

翻译成中文

2022北京租一个银行保管箱，要多少钱？ 立即咨询 广告

I agree with the new list.
Wang Lu

Re: about the author list

精简信息

发件人: tanghuixinrgg@163.com<tanghuixinrgg@163.com>

收件人: chenyurgg<chenyurgg@163.com>

时 间: 2022年08月06日 16:53 (星期六)

翻译成中文

贵重物品怎么放?银行授权网点,24h存取 立即咨询 广告

I agree with the new list.
Huixin Tang

Re:about the author list

优化阅读 | 精简信息

发件人: xumanmanrgg<xumanmanrgg@163.com>

收件人: chenyurgg<chenyurgg@163.com>

时 间: 2022年08月06日 16:54 (星期六)

翻译成中文

【升级】邮箱会员，更安全的邮箱体验，更大的存储空间，提高办公效率！ 广告

I agree with the new author list.
Manman Xu

At 2022-08-06 16:38:23, "chenyurgg" <chenyurgg@163.com> wrote:

- 隐藏引用文字 -

Hi，everyone

In the work related to galectin-3, Manman Xu and Huixin Tang supplemented the experiments according to the reviews' suggestion. So, I suggest to add Manman Xu and Huixin Tang to the list of the authors. And the new list of authors is as follows: Li Bai, Wang Lu, Shan Tang, Huixin Tang, Manman Xu, Chen Liang, Sujun Zheng, Shuang Liu, Ming Kong, Zhongping Duan, Yu Chen. Please let me know if you agree with this list.
