# Supplementary material for: Galectin-3 critically mediates the hepatoprotection conferred by M2-like macrophages in ACLF by inhibiting pyroptosis but not necroptosis signalling
Source: Cell Death Dis. 2022 Sep 8;13(9):775. doi: 10.1038/s41419-022-05181-1 (PMC9458748; doi:10.1038/s41419-022-05181-1)
Supplement: Supplementary file 5 — AJE certificate [file 41419_2022_5181_MOESM5_ESM.pdf]

Editing Certificate

This document certifies that the manuscript

Galectin-3 critically mediates the hepatoprotection conferred by M2-like macrophages in ACLF by inhibiting pyroptosis but not necroptosis signalling

prepared by the authors

Li Bai, Wang Lu, Shan Tang, Huixin Tang, Manman Xu, Chen Liang, Sujun Zheng, Shuang Liu, Ming Kong, Zhongping Duan, Yu Chen

was edited for proper English language, grammar, punctuation, spelling, and overall style by one or more of the highly qualified native English speaking editors at AJE.

This certificate was issued on **June 27, 2022** and may be verified on the AJE website using the verification code **10D0-7C38-E2A6-5979-8AE7** .

Neither the research content nor the authors' intentions were altered in any way during the editing process. Documents receiving this certification should be English-ready for publication; however, the author has the ability to accept or reject our suggestions and changes. To verify the final AJE edited version, please visit our verification page at aje.com/certificate. If you have any questions or concerns about this edited document, please contact AJE at support@aje.com.

AJE provides a range of editing, translation, and manuscript services for researchers and publishers around the world.
For more information about our company, services, and partner discounts, please visit aje.com.
